# Carbonaceous Material Modified MoO_2_ Nanospheres with Oxygen Vacancies for Enhanced Visible-Light Photocatalytic Oxidative Coupling of Benzylamine

**DOI:** 10.3390/molecules28124739

**Published:** 2023-06-13

**Authors:** Yuhong Chang, Yanxia Zhang, Tianjun Hu, Wenwen Chen, Tao Tang, Ergui Luo, Jianfeng Jia

**Affiliations:** Key Laboratory of Magnetic Molecules and Magnetic Information Materials of Ministry of Education, School of Chemistry and Materials Science of Shanxi Normal University, Taiyuan 030032, China; changyuhong@sxnu.edu.cn (Y.C.); 703042@sxnu.edu.cn (Y.Z.); hutj@sxnu.edu.cn (T.H.); chenww@sxnu.edu.cn (W.C.); 703018@sxnu.edu.cn (T.T.); luogui1991@sxnu.edu.cn (E.L.)

**Keywords:** molybdenum dioxide, oxygen vacancy, visible light photocatalysis, dioxygen activation, selective oxidation of benzylamine

## Abstract

Surface oxygen vacancy (OV) plays a pivotal role in the activation of molecular oxygen and separation of electrons and holes in photocatalysis. Herein, carbonaceous materials-modified MoO_2_ nanospheres with abundant surface OVs (MoO_2_/C-OV) were successfully synthesized via glucose hydrothermal processes. In situ introduction of carbonaceous materials triggered a reconstruction of the MoO_2_ surface, which introduced abundant surface OVs on the MoO_2_/C composites. The surface oxygen vacancies on the obtained MoO_2_/C-OV were confirmed via electron spin resonance spectroscopy (ESR) and X-ray photoelectron spectroscopy (XPS). The surface OVs and carbonaceous materials boosted the activation of molecular oxygen to singlet oxygen (^1^O_2_) and superoxide anion radical (•O_2_^−^) in selectively photocatalytic oxidation of benzylamine to imine. The conversion of benzylamine was 10 times that of pristine MoO_2_ nanospheres with a high selectivity under visible light irradiation at 1 atm air pressure. These results open an avenue to modify Mo-based materials for visible light-driven photocatalysis.

## 1. Introduction

Photocatalytic oxidative coupling of amines to imines with a green, efficient and economical process has attracted great attention owing to the essential intermediates of imines for the production of chemical and pharmaceutical agents [1,2,3]. Researchers have made enormous efforts to develop high-performance photocatalysts for an innovative imines synthetic strategy [4,5,6]. Many kinds of photocatalysts have been developed for oxidation coupling of amines, such as plasmonic metals (Au, Ag, Cu, etc.) [7,8], semiconductor oxides (TiO_2_, WO_3_, BiOCl, etc.) [9,10], carbon materials [11,12], polymers [13] and so on. Semiconductor oxides possess environmental friendliness, cost-effectiveness, and excellent capability to harvest sunlight [14,15]. Among them, molybdenum dioxide is a promising candidate for semiconductor photocatalyst due to its cheapness, chemical stability and green synthesis [16]. MoO_2_ is the rutile-type transition metallic oxide and has been widely studied due to its visible light absorption capacity and specific metallic properties, which differ from those of other oxides [17]. The existence of a large amount of free electrons makes MoO_2_ trap electrons and enhance charge separation, which is profitable for improving the catalytic performance [18]. Additionally, MoO_2_ is widely utilized in the various fields owing to acid and alkali resistance and thermal stability [19,20]. In order to enhance the photocatalytic performance of MoO_2_, the surficial state of semiconductor oxides could be adjusted by post-treatment to construct a surface defect structure, improving charge separation and the interaction between photocatalyst and reactant molecules [21].

Oxygen vacancies (OVs) have been confirmed to act as electron donors and adsorption sites and photocatalysis active sites for O_2_ adsorption, which is highly desirable for enhancing the photocatalytic performance [22,23,24]. It has been accepted that the bulk defects are the recombination centers of photo-induced electrons and holes, which is unfavorable for photocatalytic reaction [25,26]. In comparison, surface OVs play an important role not only in the extension of the photo response, but also in increasing the charge separation efficiency [27,28,29]. Semiconductor photocatalysts with rich surface OVs, such as TiO_2_, BiO_2_, MoO_3_, WO_3_, ZnO and so on, have been extensively reported [16,30,31,32,33]. BiOCl nanosheets with surface OVs resulted in abundant surface low-coordinated Bi atoms, contributing to the assembly of O_2_ molecules on the surface of photocatalyst [34]. The appropriate concentration of surface OVs on TiO_2_ nanosheets promotes the activation of O_2_ in photocatalytic oxidative of benzylamine to imine [35]. The visible-light response ability of these photocatalysts still needs to be further improved. The common strategies for introducing surface OVs into the semiconductors include thermal treatment, chemical reduction, ultraviolet irradiation and so on [25,36,37]. Generally, these methods always involved high temperature or production of hydrogen [36]. Therefore, it makes sense to develop a simple and effective method to incorporate surface OVs into photocatalysts [27,38]. In situ introduction of carbonaceous precursors can trigger a reconstruction of the oxide surface and the formation of nanocomposites with interfacial disordered regions. [39]

In this paper, carbonaceous materials modified MoO_2_ nanospheres with abundant surface OVs (MoO_2_/C-OV) were synthesized by glucose hydrothermal processes aimed at the oxidative coupling of benzylamine. The surface OVs were constructed by the reconstruction of the MoO_2_ surface caused by the in situ introduction of carbonaceous precursors after glucose hydrothermal treatment. The presence of surface OVs could effectively improve the photocatalytic transformation of benzylamine to its corresponding imines under visible light irradiation at 1 atm pressure. The morphology, surface chemical states and electronic structures of the MoO_2_/C-OV were further elucidated by a series of characterization techniques. The unsaturated sites caused by surface OVs sites strongly interacted with molecular oxygen, which could establish a photoexcited electron transport channel. The adsorption and activation of O_2_ molecules produced active ^1^O_2_ and •O_2_^−^ species with the assistance of the surface OVs and carbonaceous materials.

## 2. Results and Discussion

### 2.1. Synthesis and Characterization of the Samples

**Figure 1:**
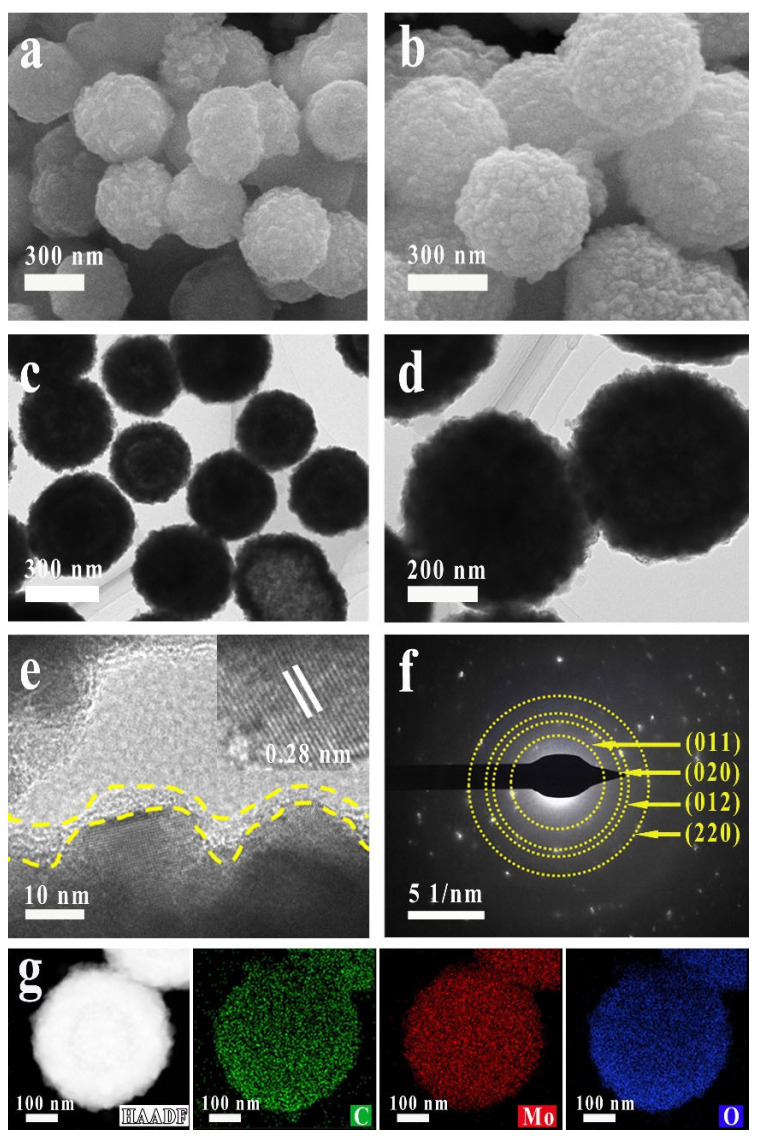
(**a**,**b**) High-magnification SEM image of the as-prepared MoO_2_/C-OV. (**c**) TEM image, (**d**) high-magnification TEM image, (**e**) HRTEM image and (**f**) SAED profiles of the as-prepared MoO_2_/C-OV. (**g**) The corresponding TEM image and EDX elemental mapping of C, Mo and O for the MoO_2_/C-OV.

The phase, morphology and crystal structure of the samples were characterized using an X-ray powder diffractometer (XRD), a scanning electron microscope (SEM), a transmission electron microscope (TEM), a high-resolution transmission electron microscope (HRTEM), selected area electron diffraction (SAED) and an X-ray energy spectrometer (EDS). The morphology of the prepared samples was analyzed via SEM and TEM. As shown in Figure S1a, MoO_2_ nanospheres with a diameter of about 500 nm consisted of a large number of tightly connected irregular nanoparticles. As shown in Figure 1a, there was no obvious change in the morphology of MoO_2_ after hydrothermal treatment with glucose. High-magnification SEM images (Figure 1b) further exhibited rough surface and sharp projections of MoO_2_ nanospheres, which would provide more surface catalytic active sites. As shown in Figure 1c,d, the self-assembled nanospheres with a rough surface are composed of many small nanoparticles approximately several tens of nanometers in size. These nanospheres are not loose aggregations of the small nanoparticles. As shown in Figure S2, the structure and morphology of MoO_2_ nanoparticles remained stable after hydrothermal and high-temperature calcination. Figure 1e displayed that the MoO_2_ nanoparticle was tightly connected with carbonaceous materials. A clear disordered area was constructed at the interface between the MoO_2_ nanosphere and carbonaceous material, revealing more active Mo atoms and providing active sites. The inset in Figure 1e is the corresponding High resolution-TEM (HRTEM), it could be confirmed that the MoO_2_ nanospheres owned clear lattice fringes. The spacing with 0.28 nm of the adjacent lattice planes was consistent with the (−102) plane of MoO_2_. As shown in Figure 1f, the characteristic polycrystalline SAED pattern showed the (011), (020), (012), (220) plane, in accordance with the XRD diffraction pattern. The diffraction ring of carbon is not clearly observed in the pattern, indicating that the carbonaceous material was in an amorphous state. As shown in the elemental mapping images (Figure 1g), a certain amount of C was uniformly distributed on the whole MoO_2_ nanosphere, expressing the successful fabrication of the MoO_2_/C-OV catalyst. The elemental mapping images of MoO_2_ and MoO_2_/C were shown in Figures S3 and S4. The results revealed that a unique interface microstructure was formed after glucose hydrothermal treatment.

**Figure 2:**
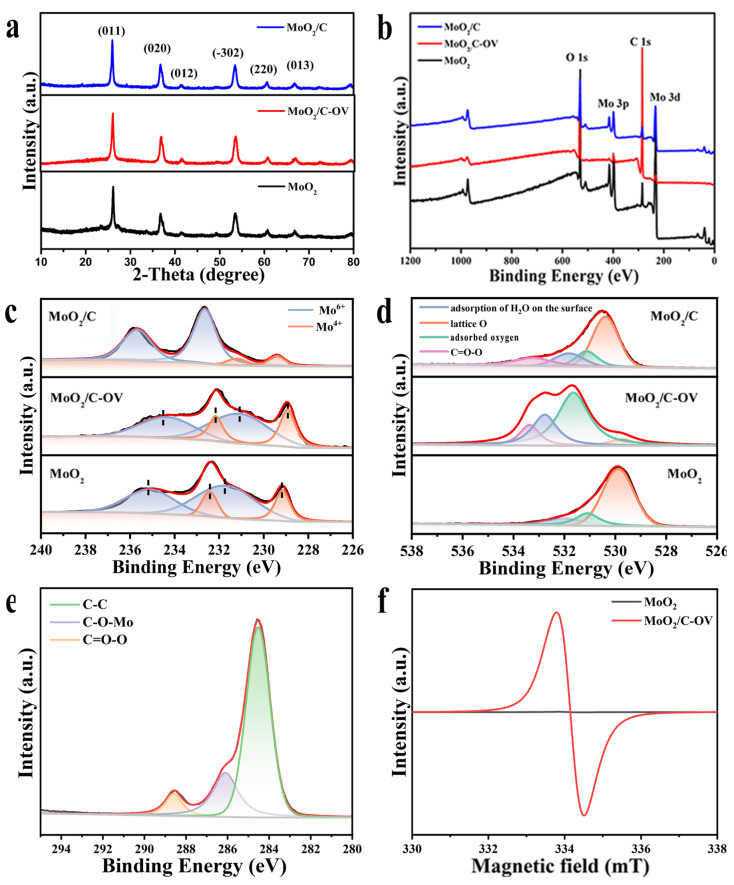
(**a**) XRD pattern, (**b**) XPS survey spectra and XPS spectra of (**c**) Mo 3d and (**d**) O 1s of MoO_2_, MoO_2_/C-OV and MoO_2_/C. (**e**) XPS spectra of C 1s in the as-prepared MoO_2_/C-OV. (**f**) Room-temperature ESR spectra of MoO_2_, MoO_2_/C-OV and MoO_2_/C.

The crystal structures of the prepared catalysts were characterized using XRD patterns. As shown in Figure 2a, the XRD pattern of MoO_2_ nanospheres revealed that the obtained sample was well indexed as the monoclinic-phase MoO_2_ (JCPDS No. 78-1069), the space group was P21/c and the cell parameters were a = 5.609 Å, b = 4.86 Å, c = 5.628 Å. Among the three common types, monoclinic MoO_2_ is the most stable structure with a deformed rutile structure. The XRD pattern exhibited the characteristic diffraction peaks at 26.1°, 37.0°, 41.5°, 49.5°, 53.5°, 60.5°, 66.7°, which are associated with (011), (020), (012), (−302), (220), (013) and (131) crystal planes. There was no change in the crystal structure of MoO_2_ after glucose hydrothermal and high-temperature calcination treatment, which shows that the oxygen vacancies did not disrupt the crystal structure. All the prepared products owned strong diffraction peaks, and no other peaks existed in the XRD pattern, which confirmed the formation of samples with high crystallinity and phase purity. The MoO_2_/C-OV and MoO_2_/C composites were synthesized successfully via a hydrothermal and calcination method using glucose as the carbon source and MoO_2_(acac)_2_ as the Mo source.

The oxygen vacancy and chemical states of the surface atoms on MoO_2_, MoO_2_/C-OV and MoO_2_/C nanospheres were further characterized via X-ray photoelectron spectroscopy (XPS). As shown in Figure 2b, the full-survey XPS spectra of the three samples displayed the copresence of Mo, O and C elements. The contents of the Mo, O, and C elements according to the XPS spectra were shown in Table S1. The contents ratios of C and Mo elements for MoO_2_/C-OV and MoO_2_/C were higher than those of MoO_2_ nanospheres, indicating that carbonaceous matter was introduced into the surface of MoO_2_ nanospheres after the treatment via a glucose hydrothermal process. As shown in Figure 2c, the deconvoluted high-resolution Mo 3d spectrum of MoO_2_ exhibited four characteristic peaks at 235.0, 232.4, 231.7 and 229.2 eV. Two peaks at 232.4 eV and 229.2 eV could be assigned to Mo 3d_3/2_ and Mo 3d_5/2_ of the Mo^4+^ state. Two weaker peaks at 235.0 eV and 231.7 eV could be assigned to Mo^6+^, illustrating slight surface oxidization due to exposure in air. For MoO_2_/C-OV sample, the binding energy of 228.9 and 232.1 eV is assigned to the low valence Mo^4+^ 3d_5/2_ and Mo^4+^ 3d_3/2_ in MoO_2_. In addition, the peaks at 231,1.0 and 234.4 eV are ascribed to the high-valence Mo^6+^ 3d_5/2_ and Mo^6+^ 3d_3/2_. After glucose hydrothermal treatment, the Mo 3d core levels presented significant shifting to lower binding energy, indicating that more electrons were transferred from carbonaceous material and a more reduced state of Mo compared to the pristine MoO_2_ nanosphere. Compared to the MoO_2_ and MoO_2_/C-OV samples, the chemical state of MoO_2_/C composite differed significantly. Two peaks of the Mo^6+^ species were observed at 235.7 and 232.7 eV; this was ascribed to the Mo 3d_3/2_ and Mo 3d_5/2_ states. Two peaks of the Mo^4+^ species were observed at 231.0 and 229.4 eV, which was ascribed to the Mo 3d_3/2_ and Mo 3d_5/2_ states [40,41,42]. This revealed the remarkable surface oxidation of MoO_2_ after high-temperature calcination treatment. The stability of oxygen vacancies is due to the adsorption of oxygen species, which is a typical feature of defect-rich oxides. As shown in Figure 2d, the deconvoluted O 1s spectrum of MoO_2_/C-OV exhibited four peaks at 529.8, 531.6, 532.8, 533.4, which could be assigned to the lattice oxygen, the surface-adsorbed oxygen or oxygen vacancies, the adsorption of H_2_O on the surface and the oxygen of C=O-C. At the same time, the characteristic peak area of the lattice oxygen is relatively small, further demonstrating that the lattice oxygen atoms have reduced and oxygen vacancies have been introduced in the MoO_2_/C-OV catalyst [43]. As listed in Table S2, the atomic percentages of each oxygen species (at%) are calculated from the XPS data of the MoO_2_, MoO_2_/C-OV and MoO_2_/C nanospheres, respectively. The results showed that the proportion of the surface-adsorbed oxygen or oxygen vacancies on the surface of MoO_2_/C-OV was higher than that of MoO_2_ and MoO_2_/C. In addition, the ratio of the lattice oxygen on the surface of MoO_2_/C-OV was about 5.4%, which was less than that of MoO_2_ and MoO_2_/C (63.4% and 52.7%). In addition, the O 1s spectrum showed the existence of C=O-C on the surface of MoO_2_/C-OV and MoO_2_/C, which further demonstrated the introduction of carbonaceous matter onto the surface of MoO_2_/C-OV and MoO_2_/C. The proportion of the oxygen species adsorbed at oxygen vacancies for MoO_2_/C-OV was about 55.3%, which was higher than that in MoO_2_ (18.7%) and MoO_2_/C (15.9%). It was further testified that many more oxygen vacancies were formed on the surface of MoO_2_/C-OV. As shown in Figure 2e, the deconvoluted C 1s spectrum showed three characteristic peaks, located at 284.5, 285.8 and 288.4 eV, belong to C−C, C−O−Mo, C=O−O [38,44,45]. It is inferred that a carbonaceous complex layer may be constructed around MoO_2_ nanospheres and combined with the MoO_2_ surface via C−O−Mo bonds after glucose hydrothermal treatment. The deconvoluted C 1s spectra of MoO_2_ and MoO_2_/C-OV were shown in Figures S4 and S5. The above results revealed that the introduction of carbonaceous precursors in situ could trigger the reconstruction and fabricate surface oxygen vacancies on the surface of MoO_2_ via a hydrothermal process.

The presence of oxygen vacancies was further confirmed via room-temperature EPR. As shown in Figure 2f, no signal was observed in the EPR spectrum of the pristine MoO_2_ nanospheres. In comparison, the obvious oxygen vacancies signal with a g value of 2.003 was exhibited for the MoO_2_/C-OV, which could be identified as unpaired electrons trapped in the oxygen vacancy [34,46]. More oxygen vacancies were generated due to the introduction of carbon atoms. The results were consistent with the XPS spectra. This demonstrated that the MoO_2_/C-OV possesses more oxygen vacancies in the surface after the glucose hydrothermal process.

**Figure 3:**
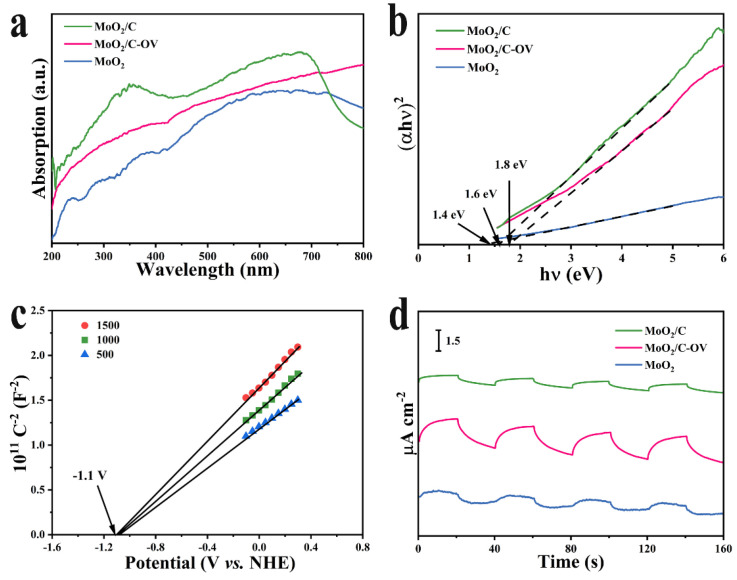
(**a**) UV-vis DRS of MoO_2_, MoO_2_/C-OV and MoO_2_/C. (**b**) The Tauc plot of MoO_2_, MoO_2_/C-OV and MoO_2_/C. (**c**) Mott–Schottky plots of MoO_2_/C-OV. (**d**) Transient photocurrent responses under visible light illumination of the samples.

The photo-response of catalysts is an important indicator of photocatalytic efficiency. The optical properties of the samples with different structures were investigated via UV-vis diffuse reflectance spectroscopy (DRS) measurement. As shown in Figure 3a, the visible-light absorption intensity of MoO_2_, especially in the near-infrared region, was significantly enhanced after the glucose hydrothermal process. MoO_2_/C-OV exhibited a powerful absorption intensity from the UV to the NIR region. The band gap energy (*E_g_*) of the photocatalysts plays an important role in evaluating the physical and optical properties, which could be calculated according to the following Formula [40].

(1) $$\alpha hv=A{\left(hv-E_{g}\right)}^{n/2}$$

where *α*, *hν*, *A* and *E_g_* represent the absorption coefficient, Planck constant, light frequency, proportionality and band gap energy, respectively. As shown in Figure 3b, MoO_2_, MoO_2_/C-OV and MoO_2_/C possessed a corresponding *E_g_* of 1.4 eV, 1.8 eV and 1.6 eV. The energy band structures of the samples were evaluated using Mott–Schottky plots, as shown in Figure 3c and Figure S7. The slope of the Mott–Schottky curve of the catalysts was positive, indicating that all samples were n-type semiconductors with electrons as the majority carriers. The indicates the flat-band potential of MoO_2_ and MoO_2_/C-OV was −0.75 V and −1.1 V vs. NHE. These results indicated that the conduction band energy of MoO_2_ and MoO_2_/C-OV was more negative than that of the O_2_/•O_2_^−^ (−0.33 V vs. NHE). O_2_ molecules adsorbed on the surface of the samples were reduced to active ∙O_2_^−^ via the photogenerated electrons, which were beneficial as they accelerated the oxidation of BA. By considering the band gap energies, the valence band energy levels (VB) of MoO_2_ and MoO_2_/C-OV were calculated to be 0.65 V and 0.7 V, respectively.

Transient photocurrent measurements were further carried out to verify the charge generation and separation efficiency, as shown in Figure 3d. Compared with the MoO_2_ and MoO_2_/C, MoO_2_/C-OV exhibited a higher photocurrent response, which suggested that the MoO_2_/C-OV would produce more photo-electrons and holes than MoO_2_ and MoO_2_/C in the same timeframe. Moreover, the separation and transfer of these generated photo-electrons and holes occurred more efficiently in the MoO_2_/C-OV than MoO_2_ and MoO_2_/C. The results demonstrated that the surface OVs would hasten the photo-electrons and holes separation to further induce the photo-electron transfer from the inside to the surface of the MoO_2_/C-OV. Therefore, it revealed that surface OVs promote the generation of the photo-electrons and accelerate the charges transfer on the interface to improve the transformation of the benzylamine molecules on the MoO_2_/C-OV.

### 2.2. Photocatalytic Activity for Oxidation of Amines to Imines

**Figure 4:**
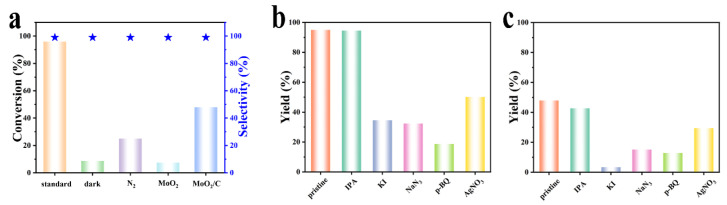
(**a**) Control experiments for photocatalytic oxidation of benzylamine to N-benzylidenebenzylamine under visible light irradiation. Effect of scavengers on the photocatalytic oxidative coupling of (**b**) MoO_2_/C-OV and (**c**) MoO_2_/C.

The photocatalytic performance of the MoO_2_ samples was evaluated via selective oxidative coupling of amines to imines under white LED light irradiation at air pressure. The oxidative coupling of BA to N-benzylidenebenzylamine was chosen as the type of reaction to explore the activation of molecular oxygen. The control experiment was run in the absence of catalyst, and there was no product under visible light irradiation for 3.5 h. As shown in Figure 4a, the conversion was only 25% with >99% selectivity when air was replaced with N_2_, demonstrating the indispensable role of molecular oxygen for the photocatalytic oxidative coupling of BA. To confirm the effect of illuminant on the oxidation reaction, the dark reaction was carried out with other things being equal. The control reaction was testified to be very slow, and the conversion was situated at 9% in the presence of MoO_2_/C-OV under the dark condition. In the presence of visible light under the same conditions, the MoO_2_/C-OV exhibited much higher conversion (96%) and selectivity (>99%) for the formation of N-benzylidenebenzylamine. The concentration of oxygen vacancy sites of MoO_2_/C sample was less than that of MoO_2_/C-OV. As the comparison, the conversion of MoO_2_/C was only 50% with >99% selectivity of N-benzylidenebenzylamine under identical conditions. The results confirmed that the presence of surface OVs was the critical requirement for the activation of molecular oxygen. The conversion of MoO_2_ (7%) was much lower than that of MoO_2_/C-OV, which indicated that the synergistic effect of carbonaceous material and oxygen vacancies played an important role in the photocatalytic reaction.

The main photo-generated active species during the photocatalytic BA oxidation were verified through radical trapping experiments with a series of scavengers over the MoO_2_/C-OV sample. As shown in Figure 4b, the conversion of BA dramatically decreased with the addition of KI (h^+^ scavenger), AgNO_3_ (e^−^ scavenger), *p*-BQ (•O_2_^−^ scavenger) or NaN_3_ (^1^O_2_ scavenger), while IPA (•OH scavenger) exhibited a negligible quenching effect [47,48,49]. The BA conversion significantly reduced with the addition of trapping agents: 34.5% (with KI), 32.4% (NaN_3_), 18.7% (*p*-BQ) and 51.9% (AgNO_3_). The results indicated that h^+^, e^−^, •O_2_^−^ and ^1^O_2_ played a key role in the reaction process for the MoO_2_/C-OV sample, where •O_2_^−^ and ^1^O_2_ are the main active species in the photocatalytic reaction. The results proved that both photo-generated holes and electrons were the initial driving forces for the photocatalytic coupling reaction. The conversions of MoO_2_/C with the addition of trapping agents were lower than that of MoO_2_/C-OV. As shown in Figure 4c, the MoO_2_/C sample has the same changing tendency as the results of the MoO_2_/C-OV sample, which implied that a similar photocatalytic process was involved. The results further revealed that the oxygen vacancy played an important role in visible light photocatalytic selective oxidation of BA. The formation of H_2_O_2_ was detected via the iodometry method in the photocatalytic oxidation of BA. The result illustrated that the H_2_O_2_, produced by the reaction of •O_2_^−^ and protons, was an intermediate in the photocatalytic aerobic oxidative coupling reaction of BA. Hence, the radical experiments indicated that h^+^, e^−^, •O_2_^−^ and ^1^O_2_ species played a key role in the photocatalytic oxidation of BA.

In order to prove the versatility of the as-prepared photocatalyst, the selective oxidation of amine derivatives was considered. The MoO_2_/C-OV sample showed a high efficiency for the visible light-driven photocatalytic oxidation of amines at 1 atm air pressure. As listed in Table 1, the arylamines with electron-withdrawing groups would be more challenging to transform into the corresponding imines than that of arylamines with electron-donating groups. It is reported that the electron-induced effect of an electron-withdrawing group could enhance the oxidative coupling of the C-N bond via the α-carbon activation. The electron inductive effect of the -Cl group is relatively weaker than that of the -F group. For Entry 2 and 3, the reactant substrate with the -F group showed high conversion under the same conditions. Entries 6 and 7, the reactant substrates with an electron-donating group, exhibited a high yield to produce their corresponding imines with high selectivity under the same reaction conditions, owing to the conjugative effect of the substituent groups. In addition, for Entries 3–5 and 7–9, the para and meta substitution of the reactant substrates was beneficial, offering greater activation of α-carbon than that of ortho substitution due to the steric effect.

### 2.3. Proposed Mechanism of Photocatalytic BA Coupling to N-benzylidenebenzylamine

**Figure 5:**
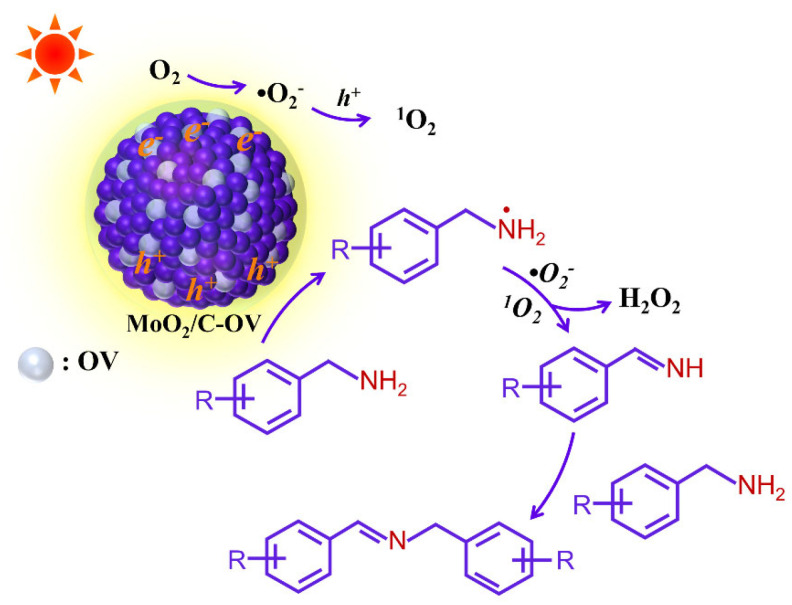
Proposed mechanism for the photocatalytic selective oxidative coupling of BA on MoO_2_/C-OV catalyst under visible light irradiation.

A reaction mechanism is proposed for the selective transformation of BA to N-benzylidenebenzylamine over MoO_2_/C-OV, as shown in Figure 5, based on the above results and discussions. The surface OVs sites of MoO_2_/C-OV would collect abundant O_2_ molecules from the air. Under the visible light irradiation, electrons and holes were generated in the MoO_2_/C-OV photocatalyst. The photogenerated electrons rapidly transferred to the positively charged surface OVs of the MoO_2_/C-OV, which played an important role in the further reduction of adsorbed O_2_ molecules to produce active •O_2_^−^ anion radicals. The holes generated transferred from bulk to surface and oxidized most of the•O_2_^−^ into ^1^O_2_. Moreover, the adsorbed BA underwent oxidation via photogenerated holes to form amine radical cations, and subsequent activation by ^1^O_2_ and •O_2_^−^ radicals promoted formation of imine intermediate (RCH=NH) and H_2_O_2_. As shown in Figure S9, the H_2_O_2_ intermediate in the process of reaction was detected via the iodometry method.

## 3. Materials and Methods

### 3.1. Chemicals and Reagents

Molybdenyl acetylacetonate (MoO_2_(acac)_2_, 97%) was purchased from Sigma-Aldrich Co., Ltd. (Shanghai, China) Ethanol. D-(+)-glucose (AR) and isopropanol (IPA) were purchased from Sinopharm Chemical Reagent Co., Ltd. (Shanghai, China). All the reagents were analytical grade and used without further purifications. Ultrapure water (18.25 MΩ·cm) was used throughout the experiments.

### 3.2. Preparation of MoO_2_ Nanospheres

MoO_2_ nanospheres were synthesized via a facile one-pot solvothermal process. Typically, 600 mg of MoO_2_(acac)_2_ was dissolved into 12 mL of water, 12 mL of ethanol and 36 mL of isopropyl alcohol via magnetic stirring. After 2 h stirring, the mixed solution was transferred into a 100 mL Teflon-lined stainless-teel autoclave at 200 °C for 10 h. After cooling to room temperature, the obtained black and blue precipitates were washed with absolute ethanol and distilled water, then dried at 60 °C overnight. Finally, the MoO_2_ nanospheres were obtained.

### 3.3. Preparation of Defective MoO_2_/C Nanospheres

In a typical synthesis process, 80 mg of MoO_2_ nanospheres and 1200 mg of glucose were added into 40 mL of ultrapure water, then stirred to obtain a homogeneous solution. The mixture was poured into a 100 mL Teflon-lined stainless-teel autoclave at 180 °C for 3 h. The precipitates were gathered via centrifugation and washed with absolute ethanol and water. Finally, the samples were marked MoO_2_/C-OV. The as-prepared MoO_2_/C-OV sample was calcined in an air flow at 300 °C to obtain MoO_2_/C.

### 3.4. Electrochemistry Measurements

Electrochemical measurements were characterized on a CH660D electrochemical workstation in a three-electrode model, using the sample films as the working electrode, saturated calomel electrode (SCE) as the reference electrode and Pt wire as the counter electrode. The working electrode was prepared as follows: 5 mg of catalyst was dispersed in 0.5 mL of ethanol and 25 μL of Nafion solution, and ultrasonically treated until a homogeneous mixture was obtained. The Mott–Schottky, electrochemical impedance spectroscopy (EIS) and photocurrent response measurements were performed at the electrochemical workstation with the working electrodes immersed in 0.1 M Na_2_SO_4_ aqueous solution.

### 3.5. Photocatalytic Oxidative Coupling of Benzylamine

The photocatalytic reactions were conducted under an air atmosphere at 45 °C for 3.5 h. The reaction system was composed of a catalyst (6 mg) and benzylamine (0.077 mmol) in acetonitrile (3 mL) using white LED light as the light source. The optical power density at the liquid surface was about 300 mW cm^−2^. The reaction products were analyzed via gas chromatography (GC, Agilent 7890B, Agilent, Santa Clara, CA, USA). The reactive oxygen species (ROS) quenching experiments Radical trapping experiments were carried out under the above conditions, except for the addition of scavengers. Isopropanol (IPA), KI, *p*-benzoquinone (*p*BQ) and NaN_3_ were used as the scavengers for hydroxyl radicals (•OH), holes (h^+^), superoxide radicals (•O_2_^−^) and singlet oxygen (^1^O_2_), respectively.

### 3.6. Detection of H_2_O_2_

The iodometry method was selected to detect the H_2_O_2_ produced in the photocatalytic reaction process. The reaction equation is as follows: $H_{2}O_{2}+3I^{-}+2H^{+}\to I^{3-}+2H_{2}O$ The H_2_O_2_ reacted with I^−^ under acidic conditions to form I^3−^, which shows strong absorption at about 350 nm. The solution A: KI (249 mg) was dissolved in ultrapure water (15 mL). Solution B: the potassium biphthalate (1.225 g) was added to 15 mL of ultrapure water. The detection process is as follows: after mixing 2 mL of solution A and 2 mL of solution B, 0.1 mL reaction solution and 0.9 mL ultrapure water were added and sonicated for 1 min. The absorption spectrum was measured using a UV-vis spectrophotometer.

## 4. Conclusions

In summary, defective MoO_2_/C nanospheres were successfully prepared via the one-pot glucose hydrothermal method. The MoO_2_ nanospheres combined closely with carbonaceous materials, forming a clear disordered region and a unique interface microstructure. In situ introduction of carbonaceous materials triggered a reconstruction of the MoO_2_ surface, which introduced abundant surface OVs on the MoO_2_/C composites. The surface OVs and carbonaceous materials played a synergistic role in the activation of molecular oxygen into ^1^O_2_ and •O_2_^−^ species. Furthermore, the surface OVs structure led to the effective separation of the photogenerated carriers. The MoO_2_/C-OV sample displayed superior photocatalytic oxidation of benzylamine under visible light irradiation at 1 atm air pressure, and the performance was 10 times that of pristine MoO_2_ nanospheres with high selectivity (>99%). This work can provide an avenue for the design of photocatalysts used for photocatalytic transformation of amines to imines by introducing oxygen vacancy of Mo-based metal oxides.

## Figures and Tables

**Table 1:**
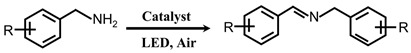
Aerobic photocatalytic oxidation of benzylamine substitutes over MoO_2_/C-OV.

Entry	Substrate	Product	Conv. (%)	Sel. (%)
1	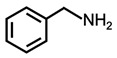	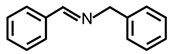	95	>99
2	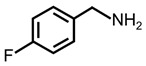	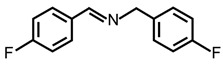	89	>99
3	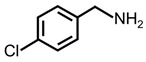	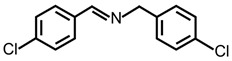	83	98
4	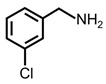	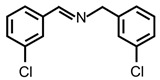	77	>99
5	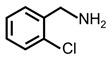	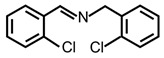	73	>99
6	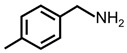	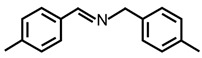	95	>99
7	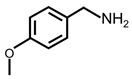	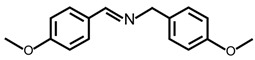	99	>99
8	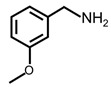	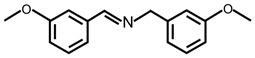	99	>99
9	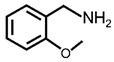	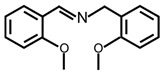	96	97

Reaction conditions: Benzylamine (0.077 mmol), catalyst (6 mg), acetonitrile (3 mL), air (1 atm), white LED light (300 mW cm^−2^), irradiation time (3.5 h).

## Data Availability

The data presented in this study are available on request from the corresponding author.

## Supplementary Materials

The following supporting information can be downloaded at: https://www.mdpi.com/article/10.3390/molecules28124739/s1, characterizations and measurement details, Figures S1–S9, Tables S1 and S2. Figure S1. SEM images of (a) MoO_2_ samples, (b) MoO_2_/C samples; Figure S2. TEM images of (a) MoO_2_ and (b) MoO_2_/C; Figure S3. The corresponding TEM image and EDX elemental mapping of Mo and O for the MoO_2_; Figure S4. The corresponding TEM image and EDX elemental mapping of C, Mo and O for the MoO_2_/C; Figure S5. XPS spectra of C 1s in the as-prepared MoO_2_; Figure S6. XPS spectra of C 1s in the as-prepared MoO_2_/C; Figure S7. Mott–Schottky plots of MoO_2_; Figure S8. EIS Nyquist plots of MoO_2_, MoO_2_/C-OV, MoO_2_/C; Figure S9. UV-vis absorption spectra of the solution after photocatalytic reaction for detection H_2_O_2_; Table S1. The contents of the Mo, O, and C elements according to the XPS spectra; Table S2. The spectrum parameters of the fitted O 1s peaks.
