# Supplementary material for: Carbonaceous Material Modified MoO2 Nanospheres with Oxygen Vacancies for Enhanced Visible-Light Photocatalytic Oxidative Coupling of Benzylamine
Source: Molecules. 2023 Jun 13;28(12):4739. doi: 10.3390/molecules28124739 (PMC10302678; doi:10.3390/molecules28124739)
Supplement: Supplementary file 1 [file molecules-28-04739-s001.zip › molecules-2437787-supplementary.pdf]

Supplementary material

Carbonaceous Material Modified MoO_2 Nanospheres with Oxygen Vacancies for Enhanced Visible-light Photocatalytic Oxidative Coupling of Benzylamine

Yuhong Chang, Yanxia Zhang, Tianjun Hu, Wenwen Chen, Tao Tang, Ergui Luo, Jianfeng Jia*

Key Laboratory of Magnetic Molecules and Magnetic Information Materials of Ministry of Education & School of Chemistry and Materials Science of Shanxi Normal University, TaiYuan 030032, China

***Corresponding author information:**

Pro. Jianfeng Jia

Key Laboratory of Magnetic Molecules and Magnetic Information Materials of Ministry of Education & School of Chemistry and Materials Science

Shanxi Normal University, Taiyuan 030032, China

Email: jiajf@dns.sxnu.edu.cn

Phone: 86-18636788078

Photocatalyst characterizations

X-ray diffraction (XRD) patterns were recorded on a D8 Advance Xray diffractometer (Bruker, Germany) with Cu Kα radiation. The morphologies and microstructures of the samples were examined using scanning electron microscope (SEM, Gemini 300, ZEISS), transmission electron microscopy (TEM, JEM-2100, JEOL) with an acceleration voltage of 100 kV, and high-resolution transmission electron microscopy (HRTEM, JEM-2100, JEOL) with an acceleration voltage of 200 kV. X-ray photoelectron spectra (XPS) were performed on the ThermoFisher VG ESCALAB 210. The pass energy of the spectrometer is 50 eV, the step energy of each spectrum is 0.100eV. In the actual XPS analysis, the internal standard method is generally used for calibration. The most commonly used method is to calibrate the binding energy of C1s (284.6 eV), the most common organic pollution carbon in vacuum systems, as a reference peak. The optical absorption properties were obtained by ultraviolet-visible diffuse reflectance spectroscopy (Lambda 750, PerkinElmer). Electron paramagnetic resonance (EPR) signals were recorded on a Bruker (A-300) spectrometer.

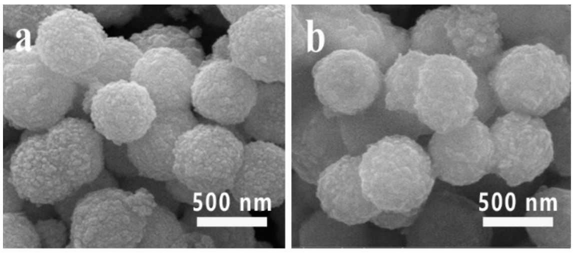

Figure S1 SEM images of (a) MoO_2 samples, (b) MoO_2/C samples.

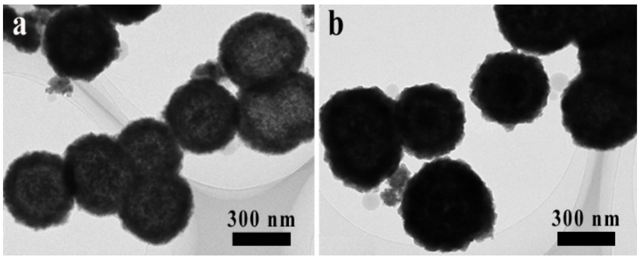

Figure S2 TEM images of (a) MoO_2 and (b) MoO_2/C

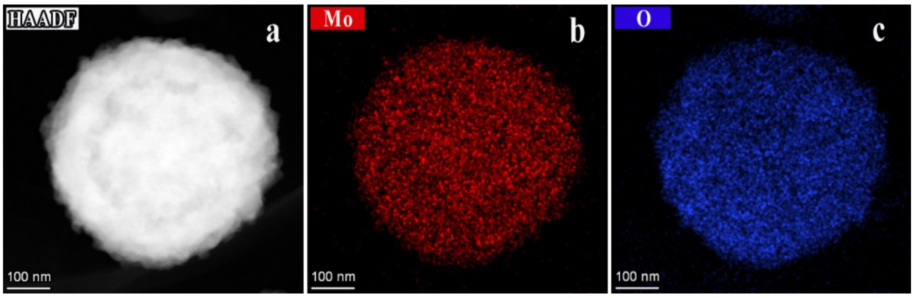

Figure S3 The corresponding TEM image and EDX elemental mapping of Mo and O for the MoO_2.

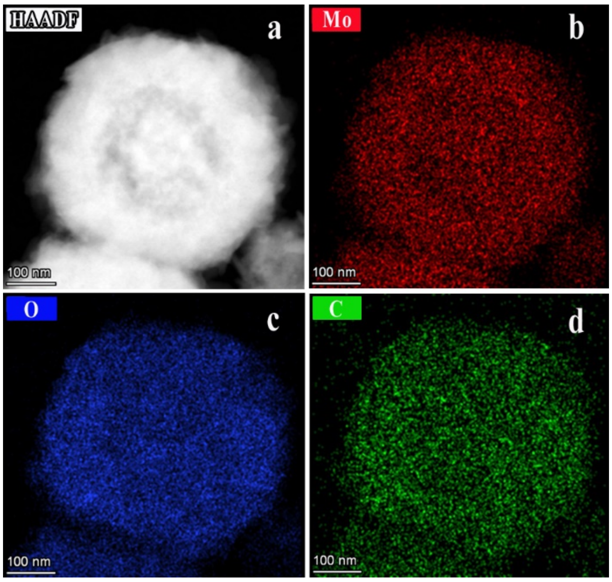

Figure S4 The corresponding TEM image and EDX elemental mapping of C, Mo and O for the MoO_2/C.

Table S1 The contents of the Mo, O, and C elements according to the XPS spectra

	O (at%)	Mo (at%)	C (at%)	Mo/C
MoO_2	34.3	52.2	13.5	3.9
MoO_2/C-OV	29.2	44.5	26.3	1.7
MoO_2/C	33.3	47.9	18.8	2.5

Table S2 The spectrum parameters of the fitted O 1s peaks.

	MoO_2		MoO_2/C-OV		MoO_2/C	
Peak	Position (eV)	at. %	Position (eV)	at. %	Position (eV)	at. %
lattice oxygen	529.8	63.4	529.8	5.4	530.3	52.7
oxygen vacancies	530.9	18.7	531.6	55.3	531.1	15.9
adsorption of H_2O on the surface	531.8	17.9	532.8	25.8	531.8	15.2
C=O-C	—	—	533.4	15.5	533.2	16.1

(page 5)

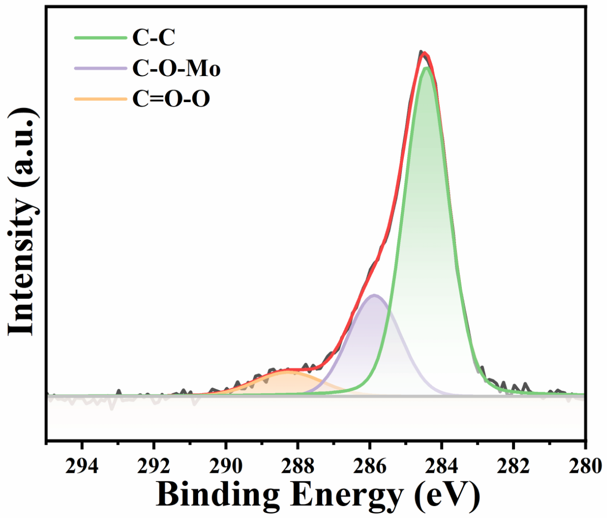

Figure S5 XPS spectra of C 1s in the as-prepared MoO_2.

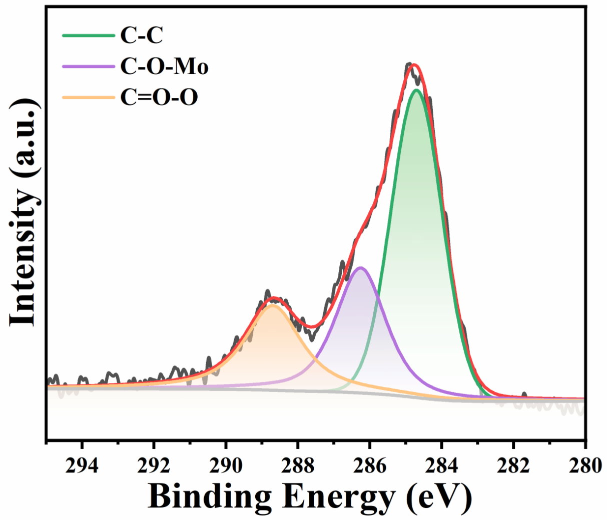

Figure S6 XPS spectra of C 1s in the as-prepared MoO_2/C.

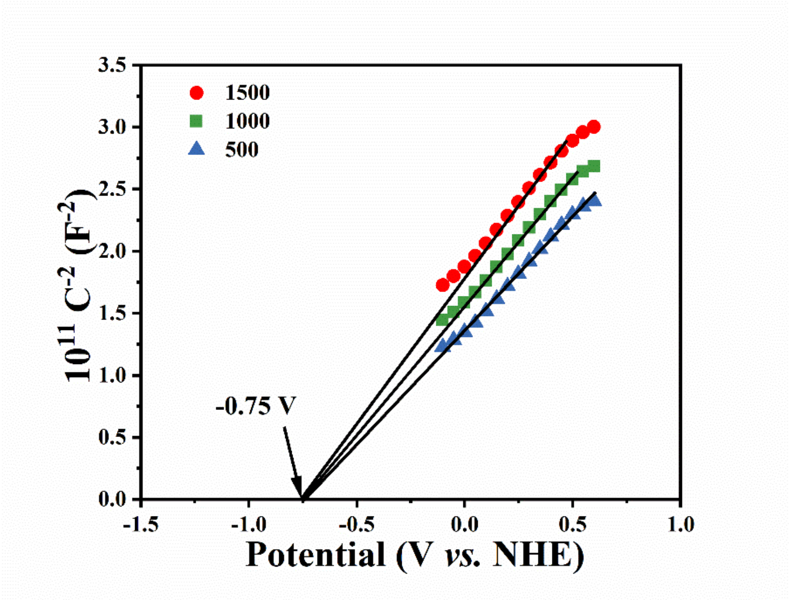

Figure S7 Mott-Schottky plots of MoO_2.

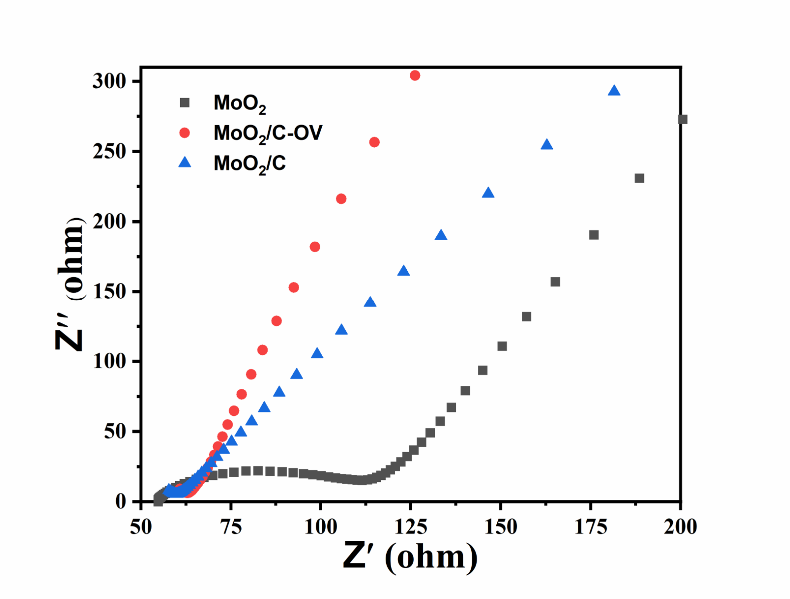

Figure S8 EIS Nyquist plots of MoO_2, MoO_2/C-OV, MoO_2/C.

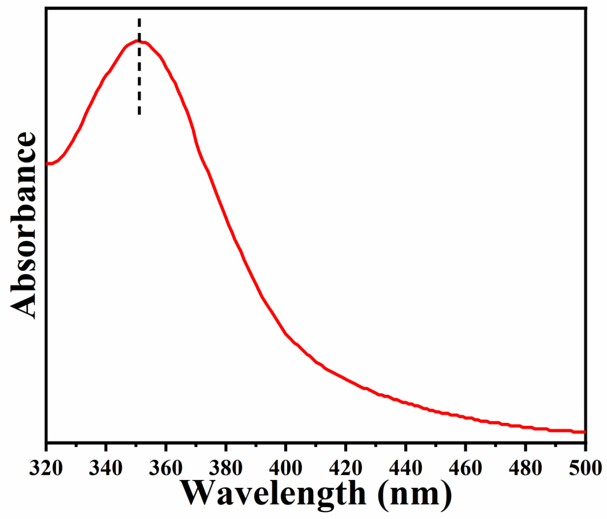

Figure S9 UV-vis absorption spectra of the solution after photocatalytic reaction for detection H_2O_2.
